# The Potential of Plant Tissue Cultures to Improve the Steviol Glycoside Profile of Stevia (*Stevia rebaudiana* Bertoni) Regenerants

**DOI:** 10.3390/ijms252413584

**Published:** 2024-12-19

**Authors:** Magdalena Dyduch-Siemińska, Karolina Wawerska, Jacek Gawroński

**Affiliations:** Department of Genetics and Horticultural Plant Breeding, Institute of Plant Genetics, Breeding and Biotechnology, University of Life Sciences in Lublin, Akademicka 15 Street, 20-950 Lublin, Poland; magdalena.dyduch@up.lublin.pl (M.D.-S.); karolina.wawerska0@gmail.com (K.W.)

**Keywords:** indirect organogenesis, in vitro culture, HPLC, micropropagation, rebaudioside A/stevioside ratio, SCoT marker, stevioside content

## Abstract

The use of in vitro cultures in plant breeding allows for obtaining cultivars with improved properties. In the case of *Stevia rebaudiana* Bert., genotypes with an appropriate rebaudioside A/stevioside ratio are desirable. The use of indirect organogenesis allows for the induction of somaclonal variation, which, consequently, results in obtaining variability within the regenerants. The Murashige and Skoog medium containing 4.0 mg × dm^−3^ 6-benzylaminopurine (BAP), 2.0 mg × dm^−3^ 1-naphthaleneacetic acid (NAA), and 2.0 mg × dm^−3^ 2,4-dichlorophenoxyacetic acid (2,4-D) resulted in obtaining plants that were biochemically and genetically diverse. The obtained regenerants were characterized by an increased content of rebaudioside A and a better rebaudioside A/stevioside ratio. Genetic analysis using SCoT (start-codon-targeted) markers showed their diversity at the molecular level. Moreover, this study showed that genotype multiplication through six subsequent re-cultures does not cause variability at the genotype level and does not affect the steviol glycoside profile. This study is the first report on obtaining genotypes with higher rebaudioside A content and a more attractive rebaudioside A to stevioside ratio through the use of in vitro cultures. The improved regenerants can be used as parents in hybridization programs or directly as valuable new genotypes.

## 1. Introduction

Stevia (*Stevia rebaudiana* Bert.) is a plant that is becoming increasingly popular due to the presence of sweet-tasting chemicals known as steviol glycosides in its leaves [1]. These compounds are excellent sugar substitutes as they are calorie-free and safe for human health, both for children and adults [2]. They were approved for use as a food additive in Europe under the code of E960 in 2011 [3]. Currently, steviol glycosides (SG) are used as sweeteners in food products and in everyday use [4,5,6]. Stevia also has antibacterial, antiseptic, anti-inflammatory, and diuretic properties [7,8]. Due to bioactive substances like tannins, carotenoids, and polyphenols, this plant can be utilized as a raw material for producing nutraceuticals and functional food through their extraction [4]. Originating in South America, stevia is now grown practically worldwide [5,9]. This shows that it adapts easily to various climate conditions, probably due to its high genetic variability [10]. Stevia is grown as a perennial plant in its area of origin. However, it is an annual plant in colder regions [11]. The aim of stevia breeding is to create genotypes characterized by high biomass production, adaptation to changing environmental conditions, and, above all, a high level of secondary metabolite production [12,13]. Generative reproduction is an easy and cheap method for commercial stevia production [9]. However, there appears to be an issue of poor seed germination in this method of plant growing. Moreover, stevia produced in this manner is not genetically and phenotypically uniform [14,15]. Clonal reproduction can be used as an alternative method by the application of various tissue culture technics. Stevia is reproduced in in vitro cultures from shoot apices and nodal explants by the technique known as microreproduction, which ensures the rapid production of large numbers of genetically stable plants [15,16]. Indirect organogenesis is a separate method for plant regeneration in in vitro cultures [17]. This process is accompanied by the formation of a callus with morphogenic properties, which creates the potential for obtaining regenerants. However, due to the callus tissue’s instability, regenerated plants can be genetically and phenotypically diverse, which is known as somaclonal variation [18]. New cultivars should have an improved steviol glycoside profile. This can be achieved with conventional breeding methods, but these are time-consuming and expensive, so the use of a biotechnological approach to creating new cultivars is particularly desirable. The use of tissue cultures will enable the implementation of the increase in the content of rebaudioside A, postulated by Dev Gautam et al. [19] as the most desirable glycoside compound in the leaves while limiting the stevioside responsible for the bitter taste. Therefore, the primary goal of this work was to obtain stevia plants regenerated by indirect organogenesis and their biochemical and genetic characterization. The regenerant genotype was analyzed using molecular markers based on the PCR—SCoT reaction. The biochemical analyses involved the determination of steviol glycoside concentration by HPLC (high-performance liquid chromatography). Furthermore, the genetic fidelity of micropropagated plants was assessed during six consecutive subcultures.

## 2. Results and Discussion

### 2.1. Stevia Regenerants Obtained by Indirect Organogenesis

#### 2.1.1. Fractionation of Steviol Glycoside Compounds by HPLC

**Figure 1:**
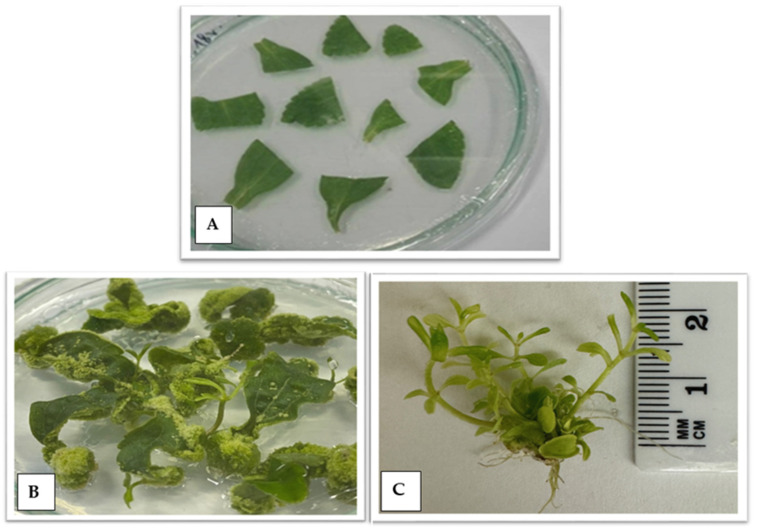
Indirect organogenesis stage—(**A**) leaf fragments placed on a petri dish; (**B**) leaf explants on a petri dish with visible callus tissue and regenerants after 6 weeks of culture; (**C**) regenerants from leaf explants after 8 weeks of culture.

**Figure 2:**
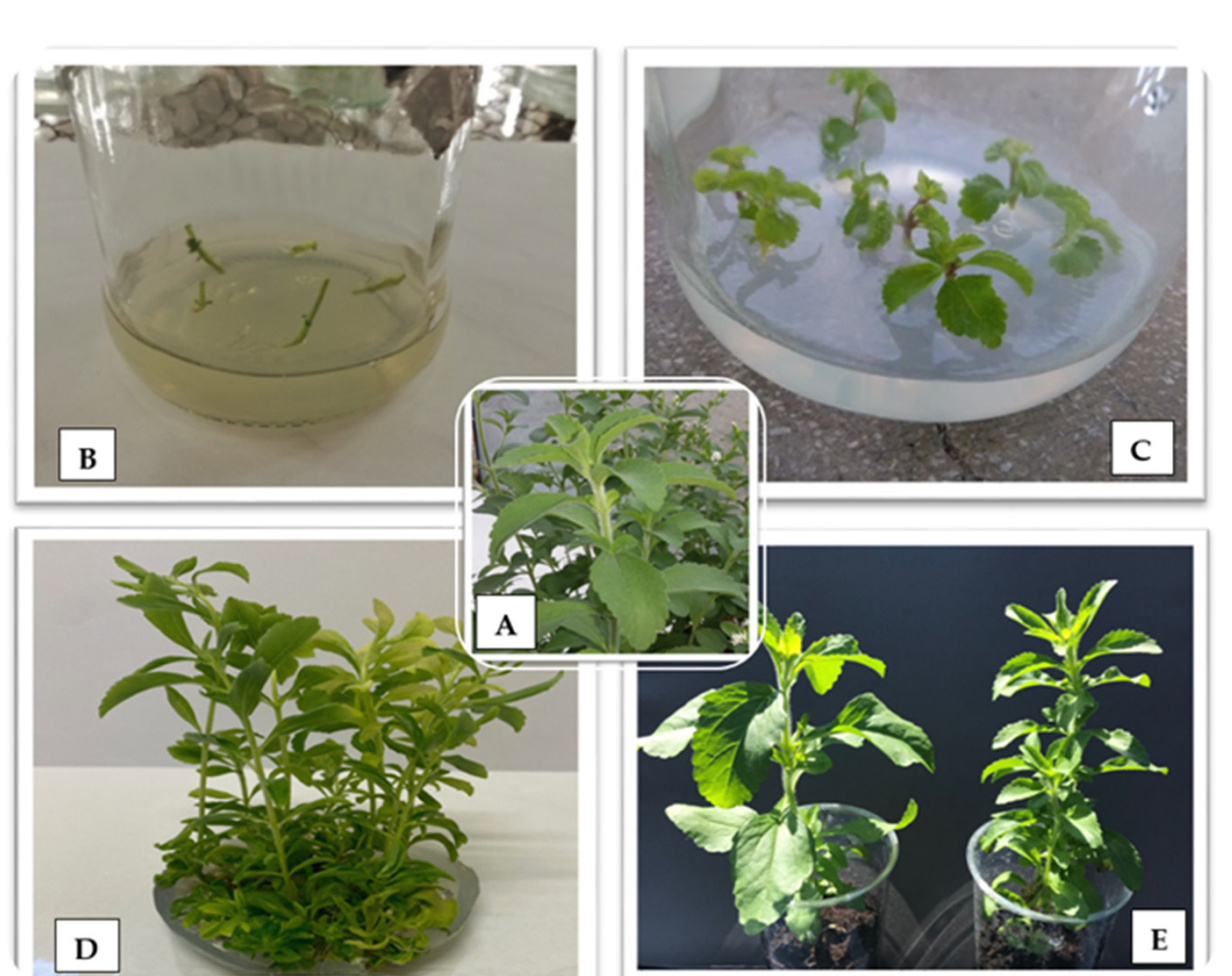
Micropropagation stages: (**A**) mother plant; (**B**) node explants; (**C**) regenerated plants after 2 weeks of culture; (**D**) culture after 6 weeks; (**E**) plants in in vivo conditions.

The most important components of stevia (*S. rebaudiana*) in terms of their technological importance include steviol glycosides, which are responsible for its sweet taste [20]. They comprise steviol connected by glycoside bonds to glucose, xylose, and rhamnose, accounting for 4 to 20% of the dry matter of stevia leaves [21]. They differ by the number of sugar units bound to the terpenoid skeleton [22]. Steviol glycosides mainly include stevioside, rebaudioside A, B, C, D, E, and F, dulcoside A, and steviolbioside [23]. According to Luwańska et al. [24], the most frequently occurring among them are stevioside and rebaudioside A and C, while the other compounds are present in much smaller amounts. In this study, the level of four steviol glycosides was analyzed, including stevioside and rebaudioside A, C, and D obtained by plant micropropagation in six consecutive passages, as well as from regenerants obtained by indirect organogenesis. The stages of indirect organogenesis are shown in Figure 1 and those of micropropagation in Figure 2. The levels of these four steviol glycosides were compared with the compound content of the mother plant. Although the sum of all the SG examined in the regenerant population varied, it was lower than the mother plant under study (Table 1). Compared with the mother plant, lower stevioside and rebaudioside C contents were found in the analyzed regenerants. Rebaudioside D content was also lower in 28 out of 30 analyzed genotypes. However, rebaudioside A content was found to have increased in 22 out of 30 regenerants under analysis, with the highest level found in regenerant numbers 6, 7, 8, 9, 15, 21, and 22. According to Yadav et al. [25] and Ghose et al. [26], the most useful extracts from stevia plants are those with higher rebaudioside A content. They report that rebaudioside A is the most highly desirable SG due to its pleasant taste, organoleptic and physicochemical properties, and higher water solubility. These characteristics are attributable to the presence of more polar groups in rebaudioside A than in stevioside. Rebaudioside A outperforms stevioside in terms of sweetness and flavor quality, especially because stevioside is commonly associated with a bitter aftertaste. Stevia breeding programs aim to improve the total glycoside content and the rebaudioside A to stevioside ratio while maintaining the high vegetative mass (leaves). The native rebaudioside A to stevioside ratio in stevia leaves is usually 0.5 or less [27]. As determined in this study, the rebaudioside A to stevioside ratio in the mother plant is very low (0.28). This indicator was found to increase in all the regenerants under analysis. The growth was more than two-fold for regenerant numbers 6, 7, 21, and 22, and it was 0.66, 0.64, 0.67, and 0.64, respectively. According to Ghose et al. [26], a change in the SG content may result from the in vitro culture conditions. Three key genes, UGT85C2, UGT74G1, and UGT76G1, are responsible for the biosynthesis of SG. The authors showed that enhanced production of stevioside and rebaudioside A in the stevia leaves of tissue-cultured plantlets directly correlated with the increased expression of the biosynthetic genes. The impact of the in vitro conditions resulting in an increase in the rebaudioside A content and a decrease in stevioside, as observed in this study, was also noticed by Yüccesan et al. [28,29] and Srivastava et al. [30]. The regeneration conditions used in our study enabled the rapid and efficient obtaining of regenerants with an improved SG profile, which would be impossible using the conventional approach based on crossing and selection.

**Figure 3:**
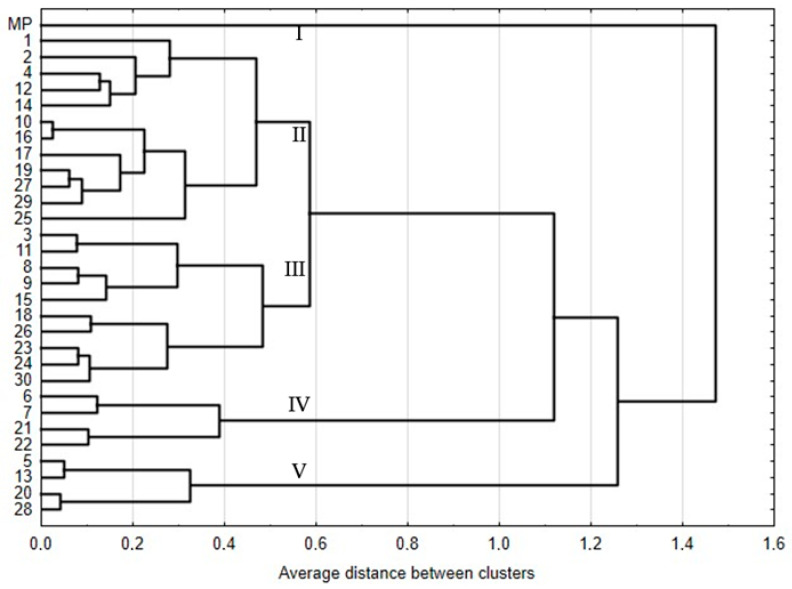
Dendrogram estimating the distance between 31 stevia genotypes based on the concentration of steviol glycosides (mg·g^−1^ dry weight).

A dendrogram was generated based on the steviol glycoside content, showing the distance between individual regenerants and the mother plant (Figure 3). It was 0.52 on average. There are five main clusters on the dendrogram. The first one includes only mother plants. This means that the steviol glycoside profile in this genotype differs from the other regenerants under analysis and confirms the presence of somaclonal variation induced in in vitro cultures during the regeneration process by indirect organogenesis. The next two largest clusters comprise 12 and 10 genotypes, respectively. The fourth cluster group regenerant numbers were 6, 7, 21, and 22, i.e., those with a beneficial rebaudioside A to stevioside ratio. The fifth cluster grouped regenerants with the lowest index value.

#### 2.1.2. Genetic Fidelity of Plants Regenerated by Indirect Organogenesis

**Figure 4:**
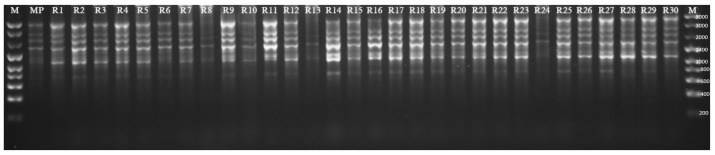
Products obtained by electrophoretic separation with the primer number 30 for 31 stevia (*S. rebaudiana*) genotypes. MP—denotes the mother plant, R1–R30—regenerants, M—size marker.

Various marker systems were used to identify the relationship between different accessions and collections of stevia both from in vivo and in vitro conditions [31,32,33,34,35,36,37]. The current study, for the first time, examined genetic diversity within a population of regenerants obtained through indirect organogenesis with tissue cultures. The experiment utilized SCoT markers, which allowed for the generation of markers associated with specific traits. The primer used in this method targets the start codon flanking region, which, according to Rai and Manoy [38], is a highly conservative region in plant genes. SCoT markers are dominant, PCR-based, and highly repetitive due to special features of a single primer (length of 18 nucleotides, high GC content (50–72%), and high annealing temperature of approximately 50 °C) [39]. Their design does not require earlier knowledge of the gene sequence, which makes this marker system usable in identifying various plant species [40,41,42]. Regenerants obtained by indirect organogenesis were also examined in this study. DNA was amplified in the analysis with 11 SCoT primers. Products obtained by electrophoretic separation with the primer number 30 (as an example) for 31 stevia (*S.rebaudiana*) genotypes are presented in Figure 4. The experiment results are listed in Table 2. The number of bands for each SCoT primer ranged from 7 to 16. The highest number of bands was observed for the SCoT 90 primer and the lowest for SCoT 4. The weights of all the obtained amplicons ranged from 300 to 7400 base pairs. Only two primers under examination (SCoT 28, SCoT 90) amplified solely monomorphic bands for all the genotypes under analysis. Polymorphic bands were generated by eight primers, i.e., SCoT: 2, 4, 21, 23, 30, 33, 75, 83. The SCoT 21 primer generated the largest number of polymorphic bands (33) (8). SCoT 23 and SCoT 46 primers generated one specific band each. The weights of the bands were 7400 and 2500 bp, respectively. The highest percentage (72.8%) of polymorphism was observed for the SCoT 21 primer, whereas the lowest was for SCoT 75 (8.3%).

Based on polymorphism identified with SCoT markers, genetic similarity between the stevia (*S.rebaudiana*) genotypes was determined according to Dice’s formula after Nei and Li [43], which ranged from 0.88 to 0.99 (Table S1). The obtained regenerants were highly similar (94%) to the mother plant. The R1 genotype was the most similar to the mother plant (99%). R13 and R28 regenerants were the least similar, with the same similarity level of 91%. The grouping of regenerant numbers 6 and 7 in one cluster, both at the phenotypic and genotypic level, characterized by the highest rebaudiosideA/stevioside ratio, indicates that genetic changes occurring during regeneration by indirect organogenesis affect the level of synthesis of these compounds. A slightly lower genetic similarity determined by the SCoT markers, at 77%, between MP and regenerants from callus in the S2 subculture was observed by Taha et al. [44] in *Salvia splendens.* However, Sharma et al. [45] demonstrated a high diversity of callus tissue relative to the starting material at 61% in *S. rebaudiana* based on RAPD markers. Since the plantlets regenerated from callus tissue in this study were replicated and subsequently analyzed individually, one should expect a higher level of genetic similarity, which was observed. This results from the fact that the regenerants were obtained from only a small part of potentially genetically diverse callus tissue cells, as noted by Kamińska et al. [46]. A genetic analysis of individual regenerants also allows for the revelation of the variability of allele composition. The highest genetic similarity observed within the regenerants was 98%. Conversely, the lowest genetic similarity of 88% was observed in R13/R27 and R13/R29.

**Table 2:** Primer sequence [39,47,48] and type of products obtained in the analysis of genotypes using SCoT markers.

No of Starter	Starter Sequence 5′-3′	Products (bp)			Polymorphism of Starter (%)
		Monomorphic	Polymorphic	Specific	
2	CAACAATGGCTACCACCC	2900; 1900; 1700; 1500	6300; 5500; 2500; 2200; 800	-	55.5
4	CAACAATGGCTACCACCT	1900; 1400; 1000	2800	-	25.0
21	ACGACATGGCGACCCACA	1600; 1400; 500	2800; 2500; 2000; 1200; 1100; 800; 700; 300	-	72.8
23	CACCATGGCTACCACCAG	2000; 1600; 900; 400	3200; 3000; 2700; 1500; 1200; 700	7400	60.0
28	CCATGGCTACCACCGCCA	2500; 1100; 900; 800; 700; 500	-	-	-
30	CCATGGCTACCACCGGCG	2200; 1700; 1600; 1400; 1100	4300; 700	-	28.6
33	CCATGGCTACCACCGCAG	3200; 2500; 1300; 500	6100; 4300; 2800; 1900; 1250; 800	-	60.0
46	ACAATGGCTACCACTGAG	3800; 3100; 2700; 1600; 1400; 1200; 1100; 900	-	2500	11.1
75	CCATGGCTACCACCGGAG	4300; 3400; 3000; 2900; 2500; 1800; 1500; 1400; 1300; 900; 750	1200	-	8.3
83	ACGACATGGCGACCAGCG	2800; 2400; 1900; 1300	5000; 1500; 800; 700	-	50.0
90	CCATGGCTACCACCGGCA	3000; 2100; 1900; 1600; 1400; 1200; 1100; 900; 800; 730; 700; 650; 580; 520; 500; 450	-	-	-
Total		68	33	2	371.3
Average		6.18	3	0.18	33.7

**Figure 5:**
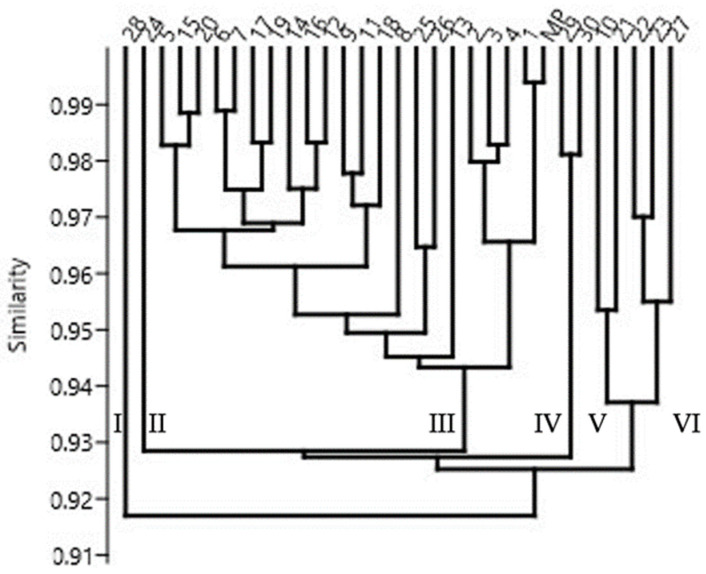
Dendrogram of the *S. rebaudiana* genotypes under study, obtained by the UPGMA method.

Figure 5 shows a dendrogram of the genotypes under study. The mean similarity level calculated for all the genotypes was 94%. Therefore, six separate clusters can be identified on the dendrogram. The first two are regenerant numbers 28 and 24. The next largest cluster consists of 22 genotypes grouped together. This also includes the mother plant, confirming that a considerable part of the regenerants under study are highly similar (Table S1). Regenerant R1 is the one that is most similar to the mother plant, indicating its closest resemblance. Cluster four comprises regenerants R29 and R30. The fifth includes regenerant numbers 10 and 21. Cluster six comprises regenerant numbers 22, 23, and 27. In other studies, due to the high genetic similarity between plantlets in the studied species, regenerants were classified into one cluster [30,49,50].

### 2.2. Stevia Regenerants Obtained by Micropropagation

#### 2.2.1. Fractionation of Steviol Glycoside Compounds by HPLC

According to the data in Table 3, the content of individual steviol glycosides is constant in consecutive re-cultures. This is consistent with the reports from Kumari and Chandra [51] on the absence of any impact of the micropropagation process on SG content. Compared with other study findings, low stevioside content in MP and plantlets in successive subcultures may result from the starting material used to establish the culture [26]. According to those authors, the SG profile variability results from the various stevia germplasms.

#### 2.2.2. Genetic Fidelity of Micropropagated Plants

**Figure 6:**
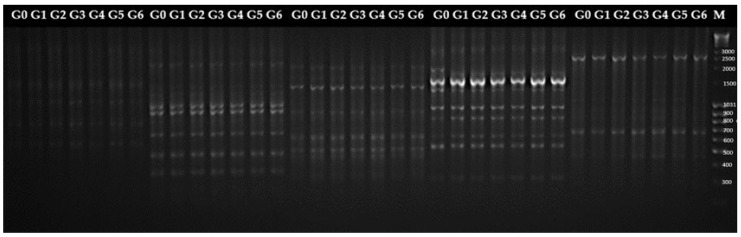
Products obtained by electrophoretic separation for primers 2, 15, 18, 19, 21. G0–G6 denote genotypes, whereas M is the size marker.

Twenty SCoT primers, whose sequence is shown in Table 4, were used in this study to analyze stevia plants from consecutive subcultures (G1–G6) and the mother plant (MP). SCoT amplification patterns of mother plants and six subcultures of *S. rebaudiana* were shown by representative gel profiles of primer 2,15,18,19, and 21 (Figure 6). Only monomorphic bands were observed, which shows that the regenerated plants are identical to MP. The total number of monomorphic amplicons was 116, with 5.8 bands per primer on average. The weight of the products obtained ranged between 250 and 6000 base pairs (bp). This is consistent with the findings presented by Clapa et al. [52], who obtained only monomorphic products, demonstrating the genetic fidelity and uniformity of stevia plants grown in vitro using the SCoT marker system. Other authors have also observed the absence of genetic variation among the tissue-culture-raised stevia plantlets. Using inter-simple sequence repeat (ISSR) markers, Thiyagarajan and Venkatachalam [53] report the absence of variability between micropropagated plants and their mother plants. Similarly, Soliman et al. [49] obtained only monomorphic bands from micropropagated stevia plants until subculture 5, also using four ISSR primers, which reflect the maintenance of allele composition during successive cycles of vegetative propagation. The current results support the views of Singh et al. [54] that direct regeneration from nodal explants showed no somaclonal variation.

## 3. Materials and Methods

### 3.1. Plant Material

This study used stevia from the experimental farm of the Department of Vegetable and Medicinal Plants, University of Life Sciences in Lublin (51°14′53″ N, 22°34′13″ E). In vitro stevia cultures were established by the procedure described by Dyduch-Siemińska [55] in the Institute of Genetics, Breeding and Biotechnology Laboratory, University of Life Sciences in Lublin.

### 3.2. Media Preparation

To prepare the Murashige and Skoog (MS) basal solid media, all stock solutions were mixed as per the prescription by Murashige and Skoog [56]. The MS basal media were supplemented with different concentrations of plant growth hormones used, respectively, for the two regeneration methods analyzed in the study—micropropagation and indirect organogenesis. All chemicals used for this work were purchased from Merck (Darmstadt, Germany). The medium pH was adjusted to 5.8 with 1N HCl and 1N NaOH. The medium was autoclaved at 121 °C for 20 min, cooled, and kept at room temperature for further use.

### 3.3. Micropropagation

The micropropagation procedure was implemented to monitor whether the tested genotype maintained stability at the biochemical and genetic level over a longer period of time covering several passages. Shoots were collected from sterile stevia plants: mother plants (MP) divided into single nodal fragments of approximately 1 cm in length and transferred into jars with 100 mL of MS medium containing 3% saccharose, 0.8% Difco bacto-agar, and 0.25 mg × dm^−3^ kinetin. The culture initiated in this way was marked as G1. In each jar, there were five nodal explants. One replication included five jars, and the experiment was conducted in three replications. The jars were kept in the culture room under controlled environmental conditions: temperature 21 ± 2 °C, 16 h light and 8 h dark conditions, approximately 3000 lux light intensity. After a 6-week culture from the reproduced plants, another five passages, labelled G2–G6, were repeated in the same time intervals, as per the methodology described above.

### 3.4. Indirect Organogenesis

Leaves were collected from sterile stevia plants, cut into 0.5 cm × 0.5 cm fragments, and placed on Petri dishes with 50 mL of MS medium containing 3% saccharose, 0.8% Difco bacto-agar, and 4.0 mg × dm^−3^ 6-benzylaminopurine (BAP), 2.0 mg × dm^−3^ 1-naphthaleneacetic acid (NAA), and 2.0 mg × dm^−3^ 2,4-dichlorophenoxyacetic acid (2,4-D). After the explants were placed on MS media, the dishes were kept in the culture room under controlled environmental conditions, as described in Section 3.3. Callus tissue was observed at the place of cutting after four weeks, and regenerants were obtained from the callus after another two weeks. Thirty randomly selected regenerants, labelled (R1–R30), were cut off and placed on a medium used for micropropagation to replicate them (the medium composition is provided in the micropropagation subchapter). The regenerant leaves were then used for genetic and biochemical analyses.

### 3.5. Genotype Analysis—SCoT Markers

An analysis of materials from micropropagation and the regenerants obtained by indirect organogenesis was performed. The plant material for DNA isolation from each of the 6 passages of the micropropagation stage consisted of samples containing leaves from 10 randomly selected microplants (plantlets) and designated G1–G6, respectively, and one sample containing plant material from the plant used to initiate the in vitro culture (MP). The leaf samples were frozen in liquid nitrogen and kept at −80 °C until the last passage was completed. Regarding the regenerant analysis (indirect organogenesis), a total of 31 genotypes were examined; 30 were labelled as R1-R30 and 1 as the mother plant (MP). The cetyl trimethyl ammonium bromide (CTAB) method described by Doyle and Doyle [57] was used to isolate genomic DNA from all samples. Start codon targeted analysis (SCoT) was performed using primers with the sequences presented in Table 2. These primers produced repeatable fragments and were selected from a group of 40 initially tested SCoT primers. A 10 µL reaction mixture consisted of water, 1×concentrated reaction buffer, 1.5 mM magnesium chloride1 µM dNTPs, 0.5 U Taq polymerase (NZYTech, Lisboa, Portugal), 0.8 µM primer (Genomed S.A., Warsaw, Poland), and 25 ng of genomic DNA. Each PCR consisted of 35 amplification cycles, including denaturation (1 min at 94 °C), primer hybridization (1 min at 50 °C), and DNA replication (2 min at 72 °C). These cycles were preceded by 3 min initial denaturation at 94 °C. Electrophoresis on a 1.5% agarose gel supplemented with 0.05 μL∙mL^−1^ ethidium bromide was carried out after each reaction. The results were visualized under UV light and photographed using a GeneSnap ver. 7.09 (SynGene, Cambridge, UK) gel documentation system. NZYDNA Ladder VIII (NZYTech Portugal) (Figure 4) and GeneRuler DNA Ladder Mix (100–10,000 bp) (Figure 6) (Thermo Scientific™) (Branchburg, NJ, USA) were used to establish the molecular weight of the products. Among the obtained SCoT products, only reproducible and clear fragments were scored from the photographs. Bands detected in analyzed genotypes and scored as present (1) or absent (0) were considered polymorphic profiles, while specific bands were restricted to a specific individual. Indistinct or weak bands were excluded from the analysis.

### 3.6. Biochemical Analysis of Regenerants by HPLC

The biochemical analysis included the mother plant (MP), plants after each consecutive re-culture (G1–G6), and regenerants (R1–R30).

#### 3.6.1. Sample Preparation and Extraction

Two grams of air-dried and crushed leaves were extracted in a Soxhlet apparatus in 150 mL of methanol for 8 h. The extract was then evaporated to dryness and diluted with 10 mL of acetonitrile. The diluted samples were centrifuged (5 min, 21,000× *g*, Universal 32, Hettich, Germany), filtered through a 0.2 µm membrane syringe filter (Costar X, Corning Inc., Salt Lake City, UT, USA), and transferred to a glass vial.

#### 3.6.2. Fractionation of Steviol Glycoside Compounds Using HPLC

Steviol glycosides were analyzed with Agilent Technologies liquid chromatograph (1200 Series) using column Phenomenex Synergi RP C18 (250 × 4.6 mm) filled with stationary phase (dp = 5 µM). UV detection was conducted at 210 nm at 28 °C and a flow rate of 0.750 mL/min. Separation was achieved in a gradient mode water: acetonitrile (A: B), 0–4 min 100–70% B, then 5–10 min 70–35% B, then returning to B in 5 min. To increase separation, 0.005% formic acid was added to both mobile phases. Stevioside, rebaudioside A, rebaudioside B, rebaudioside C, rebaudioside D, and steviol were monitored. Peak identification was based on the retention time by comparison with standard compounds.

### 3.7. Statistical Analysis of Chemical and Molecular Data

The statistical analysis of steviol glycoside concentration was carried out using ANOVA, and the significance of differences between mean values was calculated using Duncan’s multiple range tests performed at *p* < 0.05. A cluster analysis was conducted using the UPGMA (unweighted pair–group method with arithmetic mean) distance method implemented in the PAST 3 software [58]. Genetic pairwise similarities (SI-similarity index) between studied genotypes were evaluated according to Dice’s formula after Nei and Li [43] in the PAST software [58]. A cluster analysis based on steviol glycoside (mg·g^−1^ dry weight) concentration was conducted using the average linkage method available in STATISTICA 13.1. (StatSoft, Krakow, Poland).

## 4. Conclusions

This work presents the possibilities of using plant tissue cultures, both in creating new variability of stevia regenerants and in maintaining genetic identity. The technique that uses indirect organogenesis, which induces somaclonal variation, enables the selection of the most valuable regenerants in terms of the SG content. The practical use of such plants is possible, provided their genetic stability is guaranteed. This study demonstrated that the micropropagation process does not impact genetic variability and ensures that true-to-type plants can be obtained. Such plants can be used to obtain a uniform, balanced, and raw material for use in the pharmaceutical and food industries. Stevia breeding programs involve seeking genotypes containing high rebaudioside A levels. This study is the first report on obtaining genotypes with a higher rebaudioside A content and a more attractive rebaudioside A to stevioside ratio through the use of in vitro cultures. The improved regenerants can be used as parents in hybridization programs or directly as valuable new genotypes.

## Figures and Tables

**Table 1:** Concentration of steviol glycosides (mg·g^−1^ dry weight) in leaves of *S. rebaudiana* regenerants.

Genotypes	Stevioside	Rebaudioside A	RebaudiosideA/ Stevioside Ratio	Rebaudioside C	Rebaudioside D	Total
MP	5.46 ± 0.76 a^1^	1.55 ± 0.21 j–l	0.28	0.96 ± 0.10 a	0.21 ± 0.02 a–c	8.18 ± 0.94 a
1	4.94 ± 0.70 b	1.67 ± 0.24 i–k	0.34	0.48 ± 0.05 b	0.11 ± 0.01 d–f	7.20 ± 0.81 b–d
2	4.72 ± 0.60 c	1.57 ± 0.19 jk	0.33	0.41 ± 0.06 b–d	0.11 ± 0.01 d–f	6.81 ± 0.72 e–h
3	4.68 ± 0.55 c	1.97 ± 0.31 d–i	0.42	0.39 ± 0.04 b–e	0.12 ± 0.01 c–f	7.16 ± 0.80 b–e
4	4.70 ± 0.52 c	1.72 ± 0.20 h–k	0.37	0.45 ± 0.05 cbc	0.18 ± 0.02 a–d	7.05 ± 0.76 c–f
5	3.61 ± 0.41 g	1.18 ± 0.13 mn	0.33	0.25 ± 0.03 f–j	0.11 ± 0.01 d–f	5.15 ± 0.60 o
6	3.73 ± 0.38 g	2.48 ± 0.29 a	0.66	0.26 ± 0.03 f–j	0.15 ± 0.01 a–f	6.62 ± 0.63 g–j
7	3.74 ± 0.40 g	2.39 ± 0.33 ab	0.64	0.25 ± 0.03 f–j	0.23 ± 0.02 ab	6.61 ± 0.65 g–j
8	4.73 ± 0.42 c	2.24 ± 0.21 a–d	0.47	0.31 ± 0.03 d–i	0.13 ± 0.01 c–f	7.41 ± 0.69 bc
9	4.71 ± 0.60 c	2.31 ± 0.25 a–d	0.49	0.28 ± 0.03 e–j	0.12 ± 0.01 c–f	7.42 ± 0.76 bc
10	4.51 ± 0.59 d	1.53 ± 0.16 j–l	0.34	0.36 ± 0.04 b–f	0.11 ± 0.01 d–f	6.51 ± 0.59 h–k
11	4.67 ± 0.58 c	2.03 ± 0.28 c–h	0.43	0.35 ± 0.04 c–g	0.09 ± 0.01 d–f	7.14 ± 0.80 b–e
12	4.72 ± 0.53 c	1.73 ± 0.20 h–k	0.37	0.34 ± 0.04 c–g	0.12 ± 0.01 c–f	6.91 ± 0.71 d–g
13	3.61 ± 0.49 g	1.22 ± 0.14 l–n	0.34	0.28 ± 0.03 e–j	0.12 ± 0.01 c–f	5.23 ± 0.56 o
14	4.69 ± 0.61 c	1.78 ± 0.15 g–j	0.38	0.31 ± 0.03 d–i	0.24 ± 0.03 a	7.02 ± 0.68 c–f
15	4.63 ± 0.59 c–d	2.29 ± 0.24 a–d	0.49	0.35 ± 0.04 c–g	0.21 ± 0.02 a–c	7.48 ± 0.82 b
16	4.53 ± 0.52 d	1.53 ± 0.15 j–l	0.34	0.35 ± 0.04 c–g	0.12 ± 0.01 c–f	6.53 ± 0.70 h–j
17	4.33 ± 0.60 e	1.44 ± 0.16 k–m	0.33	0.30 ± 0.03 d–j	0.08 ± 0.01 ef	6.15 ± 0.69 k–n
18	4.29 ± 0.59 e	1.81 ± 0.10 f–j	0.42	0.28 ± 0.03 e–j	0.15 ± 0.02 a–f	6.53 ± 0.71 h–j
19	4.31 ± 0.46 e	1.58 ± 0.21 j–k	0.37	0.33 ± 0.03 c–h	0.12 ± 0.01 c–f	6.34 ± 0.69 i–l
20	3.31 ± 0.38 h	1.09 ± 0.11 n	0.33	0.18 ± 0.02 j	0.08 ± 0.01 ef	4.66 ± 0.50 p
21	3.42 ± 0.40 h	2.28 ± 0.26 a–d	0.67	0.19 ± 0.02 i–j	0.10 ± 0.01 df	5.99 ± 0.60 l–n
22	3.43 ± 0.29 h	2.19 ± 0.29 a–e	0.64	0.18 ± 0.02 j	0.15 ± 0.02 a–f	5.95 ± 0.62 mn
23	4.33 ± 0.60 e	2.06 ± 0.22 c–h	0.48	0.23 ± 0.02 g–j	0.09 ± 0.01 d–f	6.71 ± 0.73 f–i
24	4.32 ± 0.58 e	2.12 ± 0.24 b–f	0.49	0.21 ± 0.02 h–j	0.14 ± 0.01 b–f	6.79 ± 0.75 e–h
25	4.13 ± 0.57 f	1.41 ± 0.18 k–n	0.34	0.26 ± 0.03 f–j	0.08 ± 0.01 ef	5.88 ± 0.57 n
26	4.28 ± 0.57 e	1.86 ± 0.19 e–j	0.43	0.25 ± 0.02 f–j	0.06 ± 0.01 f	6.45 ± 0.68 h–k
27	4.33 ± 0.60 e	1.59 ± 0.21 jk	0.37	0.29 ± 0.03 d–j	0.08 ± 0.01 ef	6.29 ± 0.70 j–n
28	3.31 ± 0.46 h	1.12 ± 0.13 mn	0.34	0.21 ± 0.02 h–j	0.08 ± 0.01 l	4.72 ± 0.53 p
29	4.30 ± 0.55 e	1.64 ± 0.18 ik	0.38	0.31 ± 0.03 d–i	0.16 ± 0.02 a–e	6.41 ± 0.71 i–k
30	4.24 ± 0.59 e–f	2.10 ± 0.23 b–g	0.50	0.18 ± 0.02 j	0.14 ± 0.01 b–f	6.66 ± 0.80 g–j
Average	4.28	1.79	0.42	0.32	0.13	6.51

Values are the means ± SD. ^1^ Different letters in the same column indicate statistically significant differences (*p* < 0.05) according to Duncan’s test.

**Table 3:** Concentration of steviol glycosides (mg·g^−1^ dry weight) in leaves of *S. rebaudiana* genotypes re-cultured six times.

Genotypes	Stevioside	Rebaudioside A	RebaudiosideA/ Stevioside Ratio	Rebaudioside C	Rebaudioside D	Total
MP	5.46 ± 0.76 a^1^	1.55 ± 0.21 a	0.28	0.96 ± 0.10 a	0.21 ± 0.02 a	8.18 ± 0.94 a
G1	5.28 ± 0.69 a	1.51 ± 0.20 a	0.28	0.98 ± 0.12 a	0.23 ± 0.02 a	8.00 ± 1.05 a
G2	5.71 ± 0.63 a	1.58 ± 0.20 a	0.27	0.96 ± 0.12 a	0.22 ± 0.02 a	8.47 ± 1.10 a
G3	5.56 ± 0.68 a	1.60 ± 0.22 a	0.29	0.99 ± 0.16 a	0.24 ± 0.03 a	8.39 ± 1.08 a
G4	5.40 ± 0.66 a	1.53 ± 0.21 a	0.28	0.95 ± 0.12 a	0.200 ± 0.02 a	8.08 ± 0.99 a
G5	5.42 ± 0.69 a	1.55 ± 0.20 a	0.28	0.98 ± 0.16 a	0.21 ± 0.02 a	8.16 ± 1.03 a
G6	5.50 ± 0.69 a	1.57 ± 0.20 a	0.28	0.96 ± 0.14 a	0.22 ± 0.03 a	8.25 ± 1.10 a
Average	5.47	1.55	0.28	0.97	0.22	8.22

Values are the means ± SD. ^1^ Different letters in the same column indicate statistically significant differences (*p* < 0.05) according to Duncan’s test.

**Table 4:** Primer sequence [39,47,48] and type of products obtained in the analysis of micropropagated plants using SCoT markers.

No of Starter	Starter Sequence 5′-3′	No of Monomorphic Bands	Range of Size (bp)
Starter 2	CAACAATGGCTACCACCC	5	600–3100
Starter 4	CAACAATGGCTACCACCT	5	1050–2350
Starter 14	ACGACATGGCGACCACGC	8	460–2200
Starter 15	ACGACATGGCGACCGCGA	7	380–2200
Starter 16	ACCATGGCTACCACCGAC	7	450–2900
Starter 18	ACCATGGCTACCACCGCC	8	550–2050
Starter 19	ACCATGGCTACCACCGGC	9	350–3100
Starter 21	ACGACATGGCGACCCACA	3	500–2500
Starter 23	CACCATGGCTACCACCAG	4	350–1850
Starter 24	CACCATGGCTACCACCAT	6	550–2100
Starter 26	ACCATGGCTACCACCGTC	5	550–1700
Starter 27	ACCATGGCTACCACCGTG	4	700–2300
Starter 28	CCATGGCTACCACCGCCA	7	600–1900
Starter 30	CCATGGCTACCACCGGCG	4	650–1400
Starter 33	CCATGGCTACCACCGCAG	5	450–2200
Starter 46	ACAATGGCTACCACTGAG	6	650–6000
Starter 75	CCATGGCTACCACCGGAG	9	450–2400
Starter 83	ACGACATGGCGACCAGCG	4	250–1800
Starter 84	ACGACATGGCGACCACGT	5	750–4200
Starter 90	CCATGGCTACCACCGGCA	5	600–1700
Total		116	260–6000

## Data Availability

The data presented in this study are available on reasonable request from the corresponding author.

## Supplementary Materials

The supporting information can be downloaded at: https://www.mdpi.com/article/10.3390/ijms252413584/s1.
