# Supplementary material for: The Potential of Plant Tissue Cultures to Improve the Steviol Glycoside Profile of Stevia (Stevia rebaudiana Bertoni) Regenerants
Source: Int J Mol Sci. 2024 Dec 19;25(24):13584. doi: 10.3390/ijms252413584 (PMC11677599; doi:10.3390/ijms252413584)
Supplement: Supplementary file 1 [file ijms-25-13584-s001.zip › ijms-3349870-supplementary.pdf]

Table S1 Genetic similarity estimated among 31 stevia regenerants, based on SCoT markers calculated by Dice's coefficient.

	0	1	2	3	4	5	6	7	8	9	10	11	12	13	14	15	16	17	18	19	20	21	22	23	24	25	26	27	28	29	30
0	1	0.99	0.98	0.96	0.96	0.96	0.94	0.94	0.95	0.94	0.93	0.95	0.95	0.91	0.93	0.96	0.95	0.94	0.95	0.94	0.96	0.92	0.93	0.93	0.92	0.95	0.94	0.93	0.91	0.93	0.95
1		1	0.98	0.95	0.96	0.96	0.94	0.94	0.96	0.94	0.93	0.95	0.95	0.91	0.94	0.95	0.95	0.95	0.96	0.93	0.95	0.92	0.94	0.93	0.92	0.96	0.95	0.93	0.91	0.94	0.96
2			1.00	0.98	0.98	0.96	0.96	0.96	0.93	0.94	0.92	0.95	0.96	0.93	0.94	0.94	0.95	0.95	0.94	0.95	0.94	0.91	0.94	0.92	0.92	0.94	0.92	0.92	0.91	0.92	0.93
3				1.00	0.98	0.95	0.96	0.97	0.91	0.95	0.90	0.94	0.94	0.93	0.94	0.93	0.93	0.95	0.94	0.96	0.93	0.91	0.92	0.90	0.92	0.92	0.94	0.91	0.93	0.90	0.92
4					1.00	0.94	0.95	0.97	0.92	0.96	0.91	0.95	0.94	0.94	0.93	0.93	0.94	0.94	0.94	0.95	0.92	0.93	0.92	0.92	0.91	0.92	0.93	0.91	0.91	0.90	0.92
5						1.00	0.98	0.98	0.96	0.97	0.94	0.98	0.97	0.94	0.96	0.98	0.96	0.97	0.97	0.97	0.98	0.94	0.94	0.94	0.95	0.97	0.95	0.94	0.92	0.93	0.95
6							1.00	0.99	0.95	0.96	0.92	0.96	0.97	0.96	0.97	0.96	0.96	0.97	0.96	0.97	0.96	0.93	0.94	0.92	0.94	0.94	0.94	0.92	0.91	0.92	0.93
7								1.00	0.94	0.97	0.91	0.97	0.97	0.96	0.97	0.96	0.96	0.98	0.97	0.98	0.96	0.94	0.94	0.92	0.94	0.94	0.95	0.92	0.92	0.91	0.93
8									1.00	0.94	0.93	0.95	0.95	0.95	0.94	0.96	0.96	0.95	0.97	0.94	0.96	0.92	0.93	0.93	0.93	0.95	0.95	0.92	0.90	0.93	0.94
9										1.00	0.93	0.98	0.95	0.94	0.95	0.95	0.94	0.94	0.97	0.95	0.95	0.95	0.91	0.93	0.93	0.94	0.94	0.92	0.93	0.90	0.92
10											1.00	0.94	0.93	0.91	0.90	0.93	0.93	0.91	0.92	0.91	0.93	0.95	0.94	0.96	0.92	0.93	0.91	0.93	0.89	0.90	0.91
11												1.00	0.97	0.95	0.94	0.97	0.96	0.97	0.98	0.97	0.97	0.95	0.92	0.94	0.94	0.94	0.94	0.92	0.92	0.91	0.93
12													1.00	0.96	0.98	0.98	0.98	0.98	0.97	0.97	0.97	0.93	0.95	0.92	0.93	0.94	0.94	0.91	0.90	0.91	0.93
13														1.00	0.95	0.94	0.94	0.95	0.95	0.96	0.93	0.91	0.91	0.89	0.94	0.90	0.94	0.88	0.90	0.88	0.89
14															1.00	0.97	0.97	0.98	0.97	0.96	0.96	0.92	0.94	0.91	0.91	0.95	0.95	0.92	0.92	0.91	0.93
15																1.00	0.97	0.97	0.97	0.97	0.99	0.93	0.93	0.92	0.94	0.96	0.96	0.92	0.92	0.93	0.95
16																	1.00	0.98	0.97	0.97	0.97	0.92	0.97	0.94	0.91	0.95	0.94	0.92	0.91	0.92	0.93
17																		1.00	0.98	0.98	0.97	0.93	0.95	0.92	0.92	0.95	0.95	0.92	0.92	0.92	0.94
18																			1.00	0.96	0.97	0.95	0.94	0.93	0.92	0.95	0.97	0.92	0.93	0.92	0.94
19																				1.00	0.98	0.93	0.94	0.92	0.93	0.94	0.94	0.91	0.92	0.92	0.93
20																					1.00	0.94	0.94	0.93	0.93	0.96	0.95	0.92	0.92	0.94	0.96
21																						1.00	0.93	0.95	0.90	0.92	0.92	0.91	0.91	0.89	0.91
22																							1.00	0.97	0.90	0.96	0.93	0.95	0.90	0.93	0.94
23																								1.00	0.91	0.95	0.93	0.96	0.92	0.92	0.94
24																									1.00	0.93	0.95	0.91	0.90	0.91	0.92
25																										1.00	0.96	0.96	0.94	0.95	0.96
26																											1.00	0.93	0.93	0.93	0.95
27																												1.00	0.94	0.93	0.95
28																													1.00	0.92	0.92
29																														1.00	0.98
30																															1.0
